# Will a boom be followed by crash? A new systemic risk measure based on right-tail risk

**DOI:** 10.3389/fpsyg.2022.1104618

**Published:** 2023-02-01

**Authors:** Qing Liu, Mengxia Xu, Jinwu Xiong

**Affiliations:** ^1^Antai College of Economics and Management, Shanghai Jiao Tong University, Shanghai, China; ^2^Shanghai Advanced Institute of Finance, Shanghai Jiao Tong University, Shanghai, China; ^3^Entrepreneur Research Center, School of Business, China University of Political Science and Law, Beijing, China

**Keywords:** systemic risk, right-tail risk, extreme volatility dependence, behavioral finance, TENET-based model

## Abstract

In this study, we demonstrate that high short-term gains on the A-share market may lead to significant losses in the future and potentially cause a market catastrophe. To study the accumulation, outbreak, and cross-sector spillover process of systemic risk in the Chinese stock market, we define right-tail risk as a large rally process that may lead to left-tail losses in the future and construct a tail volatility spillover network by distinguishing between left-tail and right-tail risk. In the risk accumulation process, the market expectation bias of common shocks considerably magnifies heterogeneity risk, and in the risk outbreak and spillover processes, the greatest systemically important and systemically susceptible sectors are banking and information technology, respectively. In addition, the level of risk spillover is extremely sensitive to tail shocks and increases as tail shocks intensify. Moreover, right-tail risk has more forward-looking predictive power for left-tail risk. Apart from achieving immediate regulatory objectives, Chinese authorities must consider market expectation bias when implementing rules. Additionally, authorities want to be wary of right-tail risk, which has the potential to create serious and pervasive damage in the future if the market is allowed to be unregulated during short-term spikes.

## 1. Introduction

The purpose of measuring systemic risk is to respond more effectively to a possible financial crisis. Although it has been argued that financial crises are sudden and highly unpredictable financial market tail events (Cole and Kehoe, 2000; Chari and Kehoe, 2003; Gorton et al., 2012), typically accompanied by a recession in economic fundamentals (Kaminsky and Reinhart, 1999), the prevailing view is that financial crises can be predicted by identifying rapid asset price booms in the short term (Minsky, 1977, 1986; Kindleberger, 1978). According to several studies (Borio and Lowe, 2002; Schularick and Taylor, 2012), fast asset price growth in the short term is a valuable early warning crisis indicator that can accurately forecast financial vulnerability (Greenwood and Hanson, 2013; Baron and Xiong, 2017; López-Salido et al., 2017; Mian et al., 2017; Krishnamurthy and Li, 2020; Greenwood et al., 2022). The current literature on systemic risk focuses primarily on left-tail risk, which can lead to direct losses, as such risk can be contagious across firms, industries, and markets based on multiple channels, causing risk resonance throughout the entire financial market and negative externalities for the real economy. However, as stated previously, the volatility in the right tail of asset returns, despite being viewed as a gain in the current period, may sometimes be a very dangerous indicator of potential future losses.

We argue that left-tail risk, which results in immediate losses, is a real-time measure of the probability of a financial crisis, whereas right-tail risk, which often contains asset bubbles, is a forward-looking estimate of the probability of a financial crisis. Some behavioral finance theories can provide justification for our analysis. Prospect theory suggests that investors’ sensitivity to left-tail losses and right-tail gains is highly asymmetric (Tversky and Kahneman, 1992; Kahneman and Tversky, 2013; Barberis et al., 2021; Wang et al., 2021), and this asymmetry implies that investors’ risk preferences differ in the presence of different shocks. Herding behavior in financial markets exacerbates volatility during moments of market overheating and postbubble recessions, hence heightening financial fragility under severe market conditions (Scharfstein and Stein, 1990; Banerjee, 1992; Trueman, 1994; Clement and Tse, 2005; Tan et al., 2008; Jiang and Verardo, 2018; Din et al., 2021).

All of the aforementioned information demonstrates that left-tail risk (which entails direct losses) and right-tail risk (which indicates asset bubbles) must be examined separately. A study of systemic risk that focuses solely on left-tail risk could result in a significant underestimation of prospective dangers and, as a result, an incorrect prediction of the possibility of financial crises. In this research, we include information on right-tail risk, also known as upside risk, in evaluating systemic risk and constructing a tail volatility spillover network by differentiating between left-tail and right-tail volatility. Although upside risk is not a novel concept (Reboredo et al., 2016; Ben Ameur et al., 2020; Ali et al., 2022; Tian et al., 2022), the relationship between left-tail risk (downside risk) and right-tail risk (upside risk) has not been adequately investigated. For instance, existing research merely explored the asymmetry of these two types of risks under different circumstances but failed to describe the interplay between left-tail risk and right-tail risk; understanding this interplay is more crucial for reducing systemic risk in practice. Specifically, we are interested in the following issues: Is a right-tail risk likely to lead to a left-tail risk in the future? Or vice versa? In other words, will a boom be followed by a crash, or vice versa? Will this relationship evolve at times of crisis? In the Tail-Event driven network risk (TENET)-based model (Härdle et al., 2016), we will distinguish between left-tail volatility and right-tail volatility to investigate these issues. Recent research has utilized the TENET model to examine the tail-risk relationship between the stock market, the futures market, and the cryptocurrency market (Wang et al., 2018; Xu et al., 2021; Foglia et al., 2022; Naeem et al., 2022; Wang et al., 2022; Yousaf and Yarovaya, 2022; Yousaf et al., 2022a,b,c). This paper modifies the standard TENET model and constructs the LR-EGARCH-TENET model by integrating right-tail information to examine left-tail and right-tail risk and their interaction across sectors.

Depending on the form of risk associations, systemic risk measurements fall into two broad groups. One group consists of structured-form measurements with direct or indirect causal linkages, such as asset linkage or liquidity linkage, the causal linkages between financial sectors or institutions being viewed as the primary source of systemic risk (Cifuentes et al., 2005). Typically, structured-form measurement employs low-frequency financial reporting data or macro data to build bilateral and network models (Upper, 2011; Greenwood et al., 2015; Glasserman and Young, 2016; Duarte and Eisenbach, 2021; Barnett et al., 2022; Bernard et al., 2022), which have accurate causality inference and economic interpretation.

The other type of measurement is the reduced form. Based on market data (such as equity returns, volatilities, and CDS spreads), studies that use this type of measurement develop bilateral or network models. In general, reduced-form approaches can be categorized as “portfolio-based” measurements based on the expected shortfall (ES) framework and tail-dependence measurements based on the value-at-risk (VaR) framework. Portfolio-based assessment is intended to estimate systemic risk by analyzing the risk contribution of individual institutions to the financial system; examples of portfolio-based measurements include SES, MES (Acharya et al., 2017), SRISK (Brownlees and Engle, 2017), and CES (Banulescu and Dumitrescu, 2015). Tail-dependence measurements are derived from the nonlinear dependence of tail losses across institutions or markets; examples of tail-dependence measurements include △CoVaR (Adrian and Brunnermeier, 2016) and tail β (Hartmann et al., 2005; Straetmans et al., 2008; De Jonghe, 2010). There are additional assessments, such as the CCA (Gray and Malone, 2008), the volatility spillover network (Diebold and Yılmaz, 2014; Yang et al., 2020), and the composite index (Gray and Malone, 2008; Allen et al., 2012; Giglio et al., 2016; Nucera et al., 2016). One of the greatest advantages of reduced-form over structured-form measurements is that reduced-form measurements (which incorporate more abundant information) can characterize the dependence across financial markets or institutions in real time by using dynamic high-frequency financial market data.

We adopt the reduced-form measure where the vast majority of the literature concentrates on left-tail risk and investigate the direct systemic losses resulting from large downward fluctuations. As discussed previously, in addition to the direct losses caused by left-tail risk, short-term, extreme upward processes, despite being viewed as gains in the present moment, are very likely to result in more severe systemic losses in the future. We discover that there is a one-third chance that a significant rally in the A-share market at daily frequency would be followed by a large decline within a week, indicating that short-term upward processes may lead to heavy losses in the near future, even triggering a market crash. A strand of studies has analyzed the Chinese stock market crash (Liu et al., 2016; Fang and Bessler, 2018; Han et al., 2019; Yousaf and Hassan, 2019; Yousaf et al., 2020; Umar et al., 2021; Leippold et al., 2022). In contrast to the preceding literature, we attempt to understand the Chinese stock market crash^1^ from the perspective of right-tail risk. Moreover, one can be concerned that systemic risk is likely driven by firm-specific crash risk rather than a cross-sectoral risk contagion (Hutton et al., 2009; Kim et al., 2011; Callen and Fang, 2013, 2015; Kim and Zhang, 2016; Andreou et al., 2017; Chang et al., 2017; He et al., 2019; Lobo et al., 2020; He et al., 2021; He and Ren, 2022). To alleviate this crucial concern, we account for firm-specific crash risk in the LR-EGARCH-TENENT model, and the empirical results demonstrate that our key conclusions are robust when firm-specific crash risk is controlled. Consequently, cross-sector risk contagion continues to be the principal source of systemic risk.

We formally define the dramatic (e.g., 95%) upside asset return volatility that could lead to left-tail losses in the future as right-tail risk. To study the process of accumulation, outbreak, and spillover of systemic risk in equity markets, a tail volatility spillover network is constructed that distinguishes between left-tail and right-tail risk. The evolution of systemic risk can be divided into three stages: first, external common shocks continue to affect the system in the time dimension; second, the increasing vulnerability of the system leads to the accumulation of heterogeneous risk; and third, heterogeneous risk outbreaks across sectors culminate in the formation of systemic risk. Given the exogenous common shock, the process described above can be broken down into two parts: the effect of the common shocks on heterogeneous risk and the diffusion of heterogeneous risk.

In analyzing the effect of common shocks on the risk of heterogeneity, we find that the EGARCH model has high explanatory power. Heterogeneity risk is significantly amplified by the market expectation bias of common shocks, while shocks themselves also affect heterogeneity risk. The regulatory implication of this finding is that market expectation bias should be considered in addition to immediate regulatory objectives when designing a two-pillar regulatory framework, particularly when using strong binding instruments. Even though the policy may positively affect the market, if the policy deviates too significantly from market expectations, the policy tends to generate procyclical right-tail risk.

In the diffusion phase following the emergence of heterogeneous risks, we build a tail volatility spillover network model based on the TENET model and plot directed acyclic graphs to examine the systemic risk spillover effects among stock market sectors. The results indicate that finance is the most systemically important sector with the highest spillover risk, while information technology is the most systemically vulnerable sector with the highest input risk. We also analyze the risk contagion effect when markets are exposed to various tail shocks. Empirical evidence suggests that, first, the risk contagion effect is significantly larger during a crisis than during normal times, regardless of whether the effect is for aggregate systemic risk, left-tail risk, or right-tail risk and second, the intensity of risk contagion increases monotonically as the market is exposed to deeper tail shocks. In addition, further research indicates that right-tail risk has stronger predictive power for left-tail risk, implying that the rapid rise in asset prices over the short term is an excellent indicator of impending financial crises.

In comparison to the relevant literature, we make the following primary contributions. First, the traditional systemic risk measure is concerned primarily with left-tail risk, focusing on direct losses arising from downward fluctuations of returns (Acerbi and Tasche, 2002; Saunders and Allen, 2002; Adrian and Brunnermeier, 2016; Acharya et al., 2017; Brownlees and Engle, 2017), whereas we distinguish between left-tail and right-tail risk information and construct the LR-EGARCH. Second, in contrast to the volatility spillover literature (Diebold and Yılmaz, 2014; Brunnermeier et al., 2020; Gong, et al., 2020; Yang et al., 2020), we focus on tail volatility and identify the spillover effects of various market states. Third, unlike the literature on jump volatility (Jung and Maderitsch, 2014; Lahaye and Neely, 2020; Yang et al., 2021; Huang et al., 2022), we make two significant contributions: 1. Jump volatility includes risk information only during periods of abrupt change, whereas tail volatility in this paper also includes tail volatility information during smooth periods. 2. Jump volatility can identify volatility spillovers only in a single market state, whereas the tail volatility spillover network enables identifying risk spillover effects across multiple market states.

The remainder of the paper is organized as follows: Section 2 shows the evolution of systemic risk and constructs the tail volatility spillover network based on right-tail risk. Section 3 presents the data and the empirical results. Finally, Section 4 concludes this paper.

## 2. Tail volatility spillover network construction

We construct an LR-EGARCH-TENET model and a tail volatility spillover network by introducing right-tail risk to address the inadequacy of traditional systemic risk measurements, which focus solely on left-tail loss. The model and network are based on the observation that the market has a higher probability of triggering a crash risk in the future after an extreme short-term rise. LR represents the asymmetry of left-tail risk and right-tail risk, EGARCH describes the accumulation effect of common shocks on the heterogeneous risk of each sector in the system, and TENET describes the diffusion and contagion process in the entire system following the emergence of heterogeneous risk.

### 2.1. Market evidence of the dependence of big booms and big falls

This study analyzes the occurrence of large stock market booms and busts in terms of the value at risk (*VaR*). Assuming that $r_{t}$ is the logarithmic stock market return at time t, for the lower quantile α and upper quantile β, the market plunge and surge are defined as $r_{t}<VaR\left(\alpha \right)$ and $r_{t}>VaR\left(\beta \right)$ , respectively. If the market rallies on the Kth trading day after a large loss or gain, then we have $r_{t+K}>0$ or $r_{t+K}<0$ . The random variable $X_{t,K}=I\left(r_{t}\cdot r_{t+K}^{*}\right)$ is defined to characterize the market’s response within K trading days after a significant price increase/decrease, where $I\left(r_{t}\cdot r_{t+K}^{*}\right)$ is an indicative function defined as follows:


(1) $$I\left(r_{t}\cdot r_{t+K}^{*}\right)=\{\begin{array}{c} \;1,r_{t}\cdot r_{t+K}^{*}>0\\ -1,r_{t}\cdot r_{t+K}^{*}<0 \end{array}$$

(2) $$r_{t+K}^{*}=\{\;\begin{array}{c} \;\max\;\left(r_{t+1},r_{t+2},\ldots ,r_{t+K}\right),r_{t}<VaR\left(\alpha \right)\;\\ \min\;\left(r_{t+1},r_{t+2},\ldots ,r_{t+K}\right),r_{t}>VaR\left(\beta \right) \end{array}$$

If the market is totally efficient, the probability of predicting $X_{t,K}$ to be either 1 or − 1 based on the information available at time *t* should both be 1/2, where $E\left(X_{t,K}\right)=0$ ， $Var\left(X_{t,K}\right)=1$ . Then, using the rule of large numbers, we derive the following statistic *W*:


(3) $$W=\frac{\bar{X}-E\left(\bar{X}\right)}{\sigma /\sqrt{n}}$$

where $\bar{X}=\sum_{1}^{n}X_{t}/n$ ; in the case of large samples, W should be a standard normal distribution. If the market is perfectly efficient, W ought to be significantly zero; if the market underreacts, this underreaction demonstrates momentum effects that the market maintains its original trend and W is significantly positive; if the market overreacts, this overreaction shows reversal effects that the market retraces and W is significantly negative. In addition to focusing on the market pullback phenomenon, we focus more on the large fall after a large boom and the large boom after a large fall. In this paper, the test of whether a large decline follows a large increase is referred to as the right-tail risk test, and the right-tail sample statistic is denoted by $W_{r}$ ; the test of whether a large rally follows a large fall is called the left-tail risk test, and the left-tail sample statistic is denoted by $W_{l}$ . Let the lower and upper quartiles be 0.05 and 0.95, respectively; then, calculate the daily stock market log returns weighted by market capitalization between January 2000^2^ and December 2021, and the statistics are as follows.

Table 1 displays the correlation between stock market booms and busts and includes the number of samples where $X_{t,K}$ is either 1 or − 1, the number of samples where a large rise at moment t is followed by a large fall ( $r_{t+K}^{*}<VaR\left(\alpha \right)$ ) during [t,t+K] $X_{t+1}^{d}$ , the number of samples where a large fall at moment t is followed by a large rise ( $r_{t+K}^{*}>VaR\left(\beta \right)$ ) during [t,t+K] $X_{t+1}^{u}$ , and the statistic *W*. $X_{t,K}=1$ represents the momentum features of stock market returns during [t,t+K] after the risk event at time t, whereas $X_{t,K}=-1$ indicates the reversal characteristics. The subsequent market response to large rallies and large drops has asymmetric dynamics at different time scales, as shown in Table 1. When K=1 , the right-tail sample presents a substantial momentum trend; i.e., the sample tends to continue climbing statistically the day after a large increase. In contrast, the left-tail sample demonstrates a major reversal tendency; i.e., the sample tends to pull back and rebound the next day after a large decline. However, when K=2,3,4,5,10 , both the left-tail and right-tail samples exhibit a highly strong reversal trend, and the market retracement tendency is readily apparent following 2 trading days of a major price increase or decrease.

**Table 1:** Statistics on the relationship between big booms and big falls in the stock market.

# days	Sample	$X_{t,K}=1$	$X_{t,K}=-1$	$X_{t+K}^{d}$	$X_{t+K}^{u}$	W
K=1	Full	280	258	18	36	0.948
	Right-tail	163	106	18	0	3.475^**^
	Left-tail	117	152	0	36	−2.133^***^
K=2	Full	139	399	33	71	−11.209^***^
	Right-tail	85	184	33	0	−6.036^***^
	Left-tail	54	215	0	71	−9.816^***^
K=3	Full	79	459	56	93	−16.382^***^
	Right-tail	50	219	56	0	−10.304^***^
	Left-tail	29	240	0	93	−12.864^***^
K=4	Full	42	496	74	115	−19.548^***^
	Right-tail	29	240	74	0	−12.864^***^
	Left-tail	13	256	0	115	−14.782^***^
K=5	Full	23	515	88	130	−21.188^***^
	Right-tail	16	253	88	0	−14.450^***^
	Left-tail	7	262	0	130	−15.515^***^
K=10	Full	0	538	146	185	−23.173^***^
	Right-tail	0	269	146	0	−16.401^***^
	Left-tail	0	269	0	185	−16.370^***^

Right-tail samples refer to samples with a large rise at moment *t*. Left-tail samples refer to samples with a large fall at moment *t*.

We formally define right-tail asset bubbles that may lead to left-tail losses in the future as right-tail risk. This part concentrates on the right-tail sample statistic $W_{r}$ , which implies right-tail risk, and the statistic $X_{t,K}^{d}$ , which records a large decline after a large rise. The results in Table 1 demonstrate that there is inertia in the accumulation of right-tail risk at daily frequency. Specifically, $W_{r}$ is significantly positive at K=1 , but the risk is eventually released gradually in the following trading days. After 5 trading days, the risk is basically released, and the statistic $W_{r}$ remains relatively stable. The risk is fully released after 10 trading days, at which time the number of $X_{t,K}=1$ is 0; i.e., all samples are retraced. This result reflects the cyclical nature of risk accumulation and risk release. In addition, within a week after the large rise, the sample size of market pullbacks is 253, much larger than that of the market, which continues to rise to 16. Moreover, 88 observations in the pullback sample have a large fall ( $r_{t+K}^{*}<VaR\left(\alpha \right)$ ); i.e., approximately one-third of the observations after the large rise have a large fall within a week. Thus, if the right-tail risk of implied asset bubbles is ignored, the systemic risk will be severely underestimated. Based on the above results, this paper combines the left-tail and right-tail risk perspectives to study the systemic risk contagion effect in China’s stock market through the tail volatility spillover network.

### 2.2. Accumulation process of heterogeneous risks

The previous section provides statistics on the dependence of large market rallies and large declines, and this section will theoretically explain how common shocks affect heterogeneous risk accumulation. This paper finds that the EGARCH model (Nelson, 1991) provides better interpretability for this process. The model is shown in Equation 4–7:


(4) $$r_{i,t}=\alpha_{0}+\alpha_{1}r_{i,t-1}+u_{i,t}$$

(5) $$u_{i,t}\;|\;F_{t-1}=\sigma_{i,t}z_{i,t}$$

(6) $$z_{i,t}\sim Skewt\left(v_{i},\varphi_{i}\right)$$

**Figure 1:**
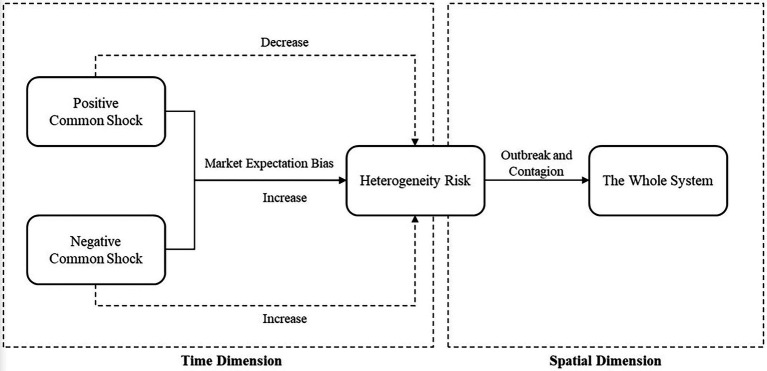
The main evolution of systemic risk.

(7) lnσi,t2=ωi+γizi,t−1−Ezi,t−1+ϕizi,t−1+βilnσi,t−12

where $r_{i,t}$ represents the logarithmic return of sector *i* at moment t . The return series is decomposed into a residual series $u_{i,t}$ through the AR(1) process. Given the previous period’s market information $F_{t-1}$ , residual $u_{i,t}$ is decomposed into the standard deviation of conditional volatility $\sigma_{i,t}$ and random variable $z_{i,t}$ . In this paper, we assume that $z_{i,t}$ represents the external common stochastic shock to sector *i*. We assume that $z_{i,t}$ obeys a skewed *t*-distribution with degrees of freedom $v_{i}$ and skewness $\varphi_{i}$ to characterize the spike and thick tail of stock market returns after a stochastic shock. $\sigma_{i,t}$ implies a heterogeneous risk shock within the system, and this heterogeneity risk can be viewed as the accumulation of different responses of sector *i* to all historical common shocks (embodied in $\gamma_{i\;\prime }\;\phi_{i}\;\mathrm{and}\;\beta_{i}$ ), with memory and agglomeration (Figure 1).

Equation 7 denotes the contagion process of systemic risk: stochastic common shocks from the outside affect the entire system, successive shocks weaken the system’s risk resilience and increase the overall vulnerability, and historical common shocks cause individual heterogeneous risks to accumulate and eventually explode; these shocks intertwine with new common shocks to induce system-wide risk spillovers. The impact mechanism of historical common shocks on heterogeneity risk consists of two parts: first, for all historical common shocks, whether positive or negative, market expectation bias ( $\left|z_{t-1}\right|-E\left[\left|z_{t-1}\right|\right]$ ) amplifies heterogeneity risk, at which time $\gamma_{i}$ , as the market expectation bias sensitivity coefficient, should theoretically be positive; second, positive and negative historical common shocks asymmetrically affect heterogeneity risk. If the positive common shock represents positive market news, its favorable component is supposed to reduce heterogeneity risk, while the negative common shock will increase heterogeneity risk. Thus, as the shock-level sensitivity coefficient, $\phi_{i}$ , should theoretically be negative. When the market faces a positive common shock, which leads to a large rise, if the heterogeneity risk increased by the market expectation deviation of the external shock is greater than that reduced by the positive part itself, a pullback or even a large fall is likely to occur, inducing a burst of right-tail risk, which in turn reflects the asset bubble bursting process of a large boom-large fall.

### 2.3. Tail volatility and conditional tail volatility

Utilizing definitions of value-at-risk (VaR) and conditional value-at-risk (CoVaR) (Saunders and Allen, 2002; Adrian and Brunnermeier, 2016), we define tail volatility (denoted as $TailVol_{i,t,\tau })\;$ as the τ quantile of conditional volatility $\sigma_{i,t}{}^{2}\;$ in the past *q* periods to describe events in which the probability of extreme conditional volatility has not exceeded τ in the past *q* periods. Unlike the traditional value-at-risk (or left-tail risk), which emphasizes potential tail losses, tail volatility focuses on extremely large volatility, including both left-tail losses and right-tail bubbles. In this paper, we argue that both left-tail expected losses generated by sharp downward volatility and right-tail asset bubbles implied by dramatic upward volatility pose significant systemic risk. Focusing solely on the left-tail risk on the loss side is insufficient to fully characterize the evolution and contagion process of systemic risk, as sharp upward fluctuations in a very short period are frequently accompanied by market collapse following the bursting of asset bubbles. Considering the asymmetry of left-tail and right-tail risks, the historical volatilities within the time window are grouped according to the positive and negative returns of the corresponding dates, and the tail volatilities and conditional tail volatilities are calculated for the left-tail and right-tail samples, respectively, as shown in Equations 8–11:


(8) $$Vol_{i,t}=\{\begin{array}{c} \begin{array}{l} Vol_{i,t}^{+}，\mathrm{all}\;\sigma_{i,t-j}^{2}\;correspondingto\;\;r_{i,t-j}\\ >0\;\mathrm{in}\;\left[t-K,t\right] \end{array}\\ \begin{array}{l} Vol_{i,t}^{-}，\mathrm{all}\;\sigma_{i,t-j}^{2}\;correspondingto\;\;r_{i,t-j}\\ <0\;\mathrm{in}\;\left[t-K,t\right] \end{array} \end{array}$$

(9) $$TailVol_{i,t,\tau }=Quantile_{\tau }\left(Vol_{i,t}\right)$$

(10) $$P\left(Vol_{i,t}\leq TailVol_{i,t,\tau }\right)|\overset{\mathrm{def}}{=}\tau$$

(11) $$P\left(Vol_{j,t}\leq CoTailVol_{j,t,\tau }|F_{i,t,\tau^{*}}\right)\overset{\mathrm{def}}{=}\tau$$

$Vol_{i,t}\;$ represents the two sets of volatilities $Vol_{i,t}^{+}\;$ and $Vol_{i,t}^{-}$ of market i within the window [t−K,t] , and $TailVol_{i,t,\tau }$ is the τ quantile of $Vol_{i,t}$ within the window. $TailVol_{i,t}^{-}\;$ represents the left-tail volatility, i.e., the tail volatility at the loss side; $TailVol_{i,t,\tau }^{+}\;$ represents the right-tail volatility, i.e., the tail volatility at the gain side (asset bubble side). $CoTailVol_{j,t,\tau }$ represents the conditional tail volatility of *j* at the τ quantile when *i* is in distress, and similarly, $CoTailVol_{i,t}^{-}$ and $CoTailVol_{i,t}^{+}\;$ represent the conditional tail volatility of the left tail and right tail, respectively. Equation 11 measures the tail volatility spillover effect on sector *j* when sector *i* is subject to a significant shock. $F_{i,t,\tau }$ represents the information set of the current period. This information set includes $TailVol_{i,t,\tau }$ , the tail volatility of sector other than *j*; $B_{j,t-1}$ , the underlying characteristics of sector *j* in the previous period; and $M_{t-1}$ , the macroeconomic indicators.

Left-tail volatility and right-tail volatility are proxy variables for left-tail loss risk and right-tail bubble risk, respectively. According to prospect theory, investors will intuitively react more to left-tail risk, which represents immediate losses, than they will to right-tail risk, which brings current gains. In other words, the market reacts asymmetrically to left-tail and right-tail risk, although asset bubbles in the right-tail may cause greater losses in the future. In addition, even if the right-tail risk implies a larger asset bubble, there is only a certain probability that the bubble will explode in the future, and the right-tail risk is not fully reflected until the bubble bursts. To avoid overestimating the systemic risk and resulting in overregulation, a risk adjustment must be made to the right-tail volatility when calculating the tail volatility $TailVol_{i,t,\tau }^{total}\;$ that represents the total systemic risk.


(12) $$TailVol_{i,t,\tau }^{total}=\frac{P^{*}}{1+P^{*}}TailVol_{i,t,\tau }^{+}+\frac{1}{1+P^{*}}TailVol_{i,t,\tau }^{-}$$

(13) $$P^{*}=P\left(r_{t+1}<VaR\left(\alpha \right)|r_{t}>VaR\left(\beta \right)\right)$$

where $P^{*}$ denotes the conditional probability of a large decline at time *t* + 1 following a large rise at time *t*. According to Table 1, approximately one-third of the observations on the Chinese stock market fell in the week following the significant increase. This paper makes these observations an exogenous variable for simplicity’s sake and uses one-third to represent the weekly conditional probability.

### 2.4. Tail volatility spillover network construction

First, we use the asymmetric slope conditional autoregressive value-at-risk model (AS-CAViaR) proposed by Engle and Manganelli (2004) to estimate the tail volatility of each sector to capture the asymmetric thick-tailed and aggregated features of return volatility:


(14) TailVoli,t,τ=θ0+θ1TailVoli,t−1,τ+θ2(ln(σi,t−12))++θ3(ln(σi,t−12))−

where $TailVol_{i,t,\tau }$ represents the left-tail volatility, right-tail volatility, and total tail volatility calculated according to Equations 9, 12. Additionally, ${\left(\ln(\sigma_{i,t-1}^{2})\right)}^{+}=\max(\ln(\sigma_{i,t-1}^{2}),0)$ and ${\left(\ln(\sigma_{i,t-1}^{2})\right)}^{-}=-\min(\ln(\sigma_{i,t-1}^{2}),0)$ represent the positive and negative log conditional volatility of sector *i* lagged by one period, respectively, to measure the asymmetric impact of positive and negative log conditional volatility on tail risk. The preceding model yields estimates of tail volatility $Tai{\overset{⌢}{lVol}}_{i,t,\tau }$ through quantile regression based on the minimum absolute deviation model.

Then, the conditional tail volatility $Co\overset{⌢}{lVol}ol^{E-TENET}$ is estimated using a single index model (Wu et al., 2010) that combines both nonlinearity and variable selection to classify the nonlinear dependence of tail volatility within the network structure.


(15) $$Vol_{j,t}=g\left(\beta_{j|F_{j,t,\tau }}^{T}F_{j,t,\tau }\right)+\varepsilon_{j,t}$$

(16) $$Co\overset{⌢}{lVol}ol^{E-TENET}\underline{\underline{\mathrm{def}}}\;Co\overset{⌢}{lVol}ol_{j|{\tilde{F}}_{j,t,\tau }}^{}{\;}^{SIM}=\hat{g}\left({\hat{\beta }}_{j|{\tilde{F}}_{j,t,\tau }}^{T}{\tilde{F}}_{j,t,\tau }\right)$$

(17) $${\hat{D}}_{j|{\tilde{F}}_{j,t,\tau }}\underline{\underline{\mathrm{def}}}\frac{\partial \hat{g}\left({\hat{\beta }}_{j|F_{j}}^{T}F_{j,t}\right)}{\partial F_{j,t}}{|}_{F_{j,t,\tau }={\tilde{F}}_{j,t,\tau }}={\hat{g}}^{\prime }\left({\hat{\beta }}_{j|{\tilde{F}}_{j}}^{T}{\tilde{F}}_{j,t,\tau }\right){\hat{\beta }}_{j|{\tilde{F}}_{j,t,\tau }}^{T}$$

(18) $$\Delta {\hat{D}}_{j|{\tilde{F}}_{j,t,\tau }}={\hat{D}}_{j|{\tilde{F}}_{j,t,\tau }}-{\hat{D}}_{j|{\tilde{F}}_{j,t,0.5}}$$

According to Equation 15, $Vol_{j,t}\;$ represents the conditional volatility $\sigma_{j,t}^{2}$ of sector *j* at time *t*; g(⋅) represents a nonlinear function with unknown form; $F_{j,t,\tau }\;\underline{\underline{\mathrm{def}}}\;\left\{Vol_{-j,t,\tau },M_{t-1},B_{j,t-1}\right\}$ represents this market state (*τ* quantile) under the information set, including the volatility $Vol_{-j,t,\tau }$ of other subjects except *j*, the macroeconomic state indicator $M_{t-1}$ of the previous period and its own basic characteristics $B_{j,t-1}$ ; $\beta_{j|F_{j,t,\tau }}\underline{\underline{\mathrm{def}}}{\left\{\beta_{j|-j},\beta_{j|M},\beta_{j|B_{j}}\right\}}^{T}$ represents the factor loadings of different information; and $\varepsilon_{j,t}$ represents the random perturbation term. In Equation 16, ${\tilde{F}}_{j,t,\tau }\;\underline{\underline{\mathrm{def}}}\left\{\hat{TailV}ol_{-j,t,\tau },M_{t-1},B_{j,t-1}\right\}$ , $\hat{TailV}ol_{-j,t,\tau }$ represents the tail volatility estimates of the other subjects except *j* when the market state is in the *τ* quantile, *and* $CoTa\hat{ilVo}l_{j|{\tilde{F}}_{j,t,\tau }}{}^{SIM}$ refers to the conditional tail volatility estimated based on the information set ${\tilde{F}}_{j,t,\tau }$ with SIM.

After the conditional tail volatility is calculated, the next step is to calculate the tail-risk spillover effect. In Equation 17, ${}_{\hat{D}j|{\tilde{F}}_{j}}$ is the gradient measure that represents the marginal impact among the covariates when $F_{j,t,\tau }={\tilde{F}}_{j,t,\tau }$ , including ${\hat{D}}_{j|{\tilde{F}}_{j,t,\tau }}\underline{\underline{\mathrm{def}}}{\left\{{\hat{D}}_{j|-j},{\hat{D}}_{j|M},{\hat{D}}_{j|B_{j}}\right\}}^{T}$ . This indicator reflects the tail volatility spillover effects in different market states, and the financial system’s tail-risk spillover network can be constructed using the aforementioned indicators. In addition, this framework permits this paper to further investigate the differences in risk spillover effects resulting from various market states. For instance, Equation 18 represents the distinction between tail-risk spillover effects under the quantile and under the median.

Among the gradient measures ${\hat{D}}_{j|{\tilde{F}}_{j,t,\tau }}$ , we examine mainly the dependence of tail volatility ${\hat{D}}_{j|-j}$ across sectors. Similar to the CoVaR model of Adrian and Brunnermeier (2016), ${\hat{D}}_{j|-j}$ more accurately represents the tail-risk spillover effects in the network structure. For example, ${\hat{D}}_{j|i}$ measures the conditional tail volatility spillover to j when sector i is subject to a significant shock, while ${\hat{D}}_{j|system}$ measures the tail volatility spillover to j when the system is distressed, and ${\hat{D}}_{system|j}$ represents the risk contribution to the entire financial system when j is distressed. The conditional value-at-risk model constructed by Adrian and Brunnermeier (2016) assumes linear dependence among covariates, whereas in real markets, return and volatility spillovers among assets are often nonlinearly asymmetric. Consequently, the LR-EGARCH-TENET model developed in this paper based on the TENET framework permits nonlinear dependence between covariates under distinct left- and right-tail risk shocks by computing ${\hat{D}}_{j|-j}$ to obtain the following matrix:


(19) $$\begin{array}{l} \;\;\;J_{1}\;\;\;\;J_{2}\;\;\;\;J_{3}\;\;\;\;\;\ldots \;\;\;\;J_{k}\\ \begin{array}{c} I_{1}\\ I_{2}\\ A_{t}=I_{3}\\ \vdots \;\\ I_{k} \end{array}\;\left[\begin{array}{ccccc} 0 & {\left({\hat{D}}_{1|2}\right)}^{+} & {\left({\hat{D}}_{1|3}\right)}^{+} & \cdots & {\left({\hat{D}}_{1|k}\right)}^{+}\\ {\left({\hat{D}}_{2|1}\right)}^{+} & 0 & {\left({\hat{D}}_{2|3}\right)}^{+} & \cdots & {\left({\hat{D}}_{2|k}\right)}^{+}\\ {\left({\hat{D}}_{3|1}\right)}^{+} & {\left({\hat{D}}_{3|2}\right)}^{+} & 0 & \cdots & {\left({\hat{D}}_{3|k}\right)}^{+}\\ \vdots & \vdots & \vdots & \ddots & \vdots \\ {\left({\hat{D}}_{k|1}\right)}^{+} & {\left({\hat{D}}_{k|2}\right)}^{+} & {\left({\hat{D}}_{k|3}\right)}^{+} & \cdots & 0 \end{array}\right] \end{array}$$

$A_{t}$ is a nondiagonal k×k sparse matrix, which measures the nonlinear dependence of tail volatility across the financial system. Each element of the matrix ${\left({\hat{D}}_{j|i}\right)}^{+}=\max\left({\hat{D}}_{j|i},0\right)$ represents the tail-risk spillover effect of sector *i* on *j*. Unlike Härdle et al. (2016), we measure the positive tail volatility spillover effect by substituting ${\left({\hat{D}}_{j|i}\right)}^{+}$ for the absolute value of the original mean spillover. The sum of each row, $I_{j,t}$ , denotes the level of total risk input at sector *j* at time *t* to assess the sector’s systemic vulnerability. The sum of each column $J_{j,t}$ represents the total risk spillover level of sector *j* at that time, thereby determining the sector’s systemic importance. The sum of all elements of the matrix $SysRisk_{t}$ represents the total systemic risk spillover level at time *t*. A unique $A_{t}$ is obtained for each moment with a fixed time window. To analyze the accumulation and evolution of systemic risk in the time dimension, we employ rolling window estimation, where the weekly frequency data are estimated on a rolling basis over a one-year window (approximately 52 weeks). That is, the estimation of each time point requires the use of data from the past 52 observations. For panels with fewer than 52 periods of observations, we use static estimation. Then, we obtain a time series of the level of risk inputs, the level of risk spillovers, and the level of total systemic risk spillovers for each subject at both the stock market and sector levels.

## 3. Empirical analysis based on the tail volatility spillover network

In the empirical analysis section, this paper investigates the tail volatility spillover effects and contemporaneous causality in the stock market at the sector level by using the LR-EGARCH-TENET model and directed acyclic graphs (DAG).

### 3.1. Data and summary statistics

In this paper, we select the front-weighted daily closing prices of A-share listed companies between January 2000 and March 2022 and then calculate the weekly log returns of each sector weighted by market capitalization based on the 11 primary sectors, which are classified by Wind industry classification, with a total of 987 observations after excluding missing values. This paper selects the one-period lagged US SP500 index, United Kingdom FTSE 100 index, French CAC40 index, German DAX30 index, Japanese Nikkei 225 index, and Chinese Hong Kong Hang Seng index as the macro control variables $M_{t-1}$ . Additionally, we select the one-period lagged sector market capitalization, price-to-earnings ratio, quick ratio (current assets/current liabilities), inventory-to-income ratio, return on net assets, and dividend distribution ratio as sector control variables $B_{t-1}$ . In addition, we control for firm-specific crash risk to exclude the impact of a single heterogeneous risk (Hutton et al., 2009; Kim et al., 2011; He et al., 2019, 2021; He and Ren, 2022). We use *crashrisk* to measure firm-specific risk, which equals 1 if a firm experiences a weekly return falling 3.2 standard deviations below the average weekly return for a fiscal year.^3^ The above data were obtained from the CSMAR database and Wind database.

Table 2 shows the conditional volatilities calculated for the sector return series after the AR(1)-EGARCH (1,1) model. As Table 2 shows, the conditional volatilities of all sectors show a very significant right skewed trend, which indicates that very large volatilities driven by tail events are common in the volatility distribution. The Jarque–Bera test shows that the distribution of conditional volatility differs remarkably from the normal distribution in all sectors, and approximately 54.5% of the sectors have a kurtosis greater than 3, especially in the financial and real estate sectors, where the kurtosis reaches 130.5 and 53.7, respectively, indicating that the variance of sector conditional volatility is very much influenced by extreme values. Therefore, separate modeling for tail volatility is needed to describe the tail volatility spillover effects under extreme tail-event shocks.

**Table 2:** Descriptive statistics of conditional volatility of 11 sectors.

Sector	Mean	Std.	Min	Median	Max	Skewness	Kurtosis
IT	0.0439	0.0119	0.0240	0.0417	0.1090	1.8646	5.8724
Utility	0.0330	0.0137	0.0133	0.0289	0.0886	1.4167	1.8433
Health care	0.0381	0.0129	0.0173	0.0360	0.0882	1.1756	1.6282
Optional consumption	0.0373	0.0128	0.0186	0.0340	0.0888	1.4423	2.0268
Industry	0.0366	0.0126	0.0180	0.0334	0.0951	1.5823	3.0567
Real estate	0.0021	0.0045	0.0000	0.0006	0.0603	5.9942	53.7778
Daily consumption	0.0014	0.0028	0.0000	0.0005	0.0283	4.7403	30.2361
Material	0.0400	0.0132	0.0201	0.0366	0.0935	1.4423	2.0143
Telecom	0.0445	0.0132	0.0226	0.0408	0.0848	0.8528	−0.1352
Energy	0.0014	0.0030	0.0000	0.0004	0.0312	4.9389	31.4296
Finance	0.0013	0.0032	0.0000	0.0004	0.0602	8.9879	130.5011

### 3.2. Parameter estimation of the EGARCH model

Table 3 shows the results of parameter estimation for each sector according to the EGARCH model, which is used mainly to describe the continuous accumulation of external common shocks and the positive feedback accumulation and outbreak of heterogeneity risk. According to Equation 7, the impact of common shocks on heterogeneity risk takes two paths. First, the market expectation bias of common shocks theoretically causes the accumulation of heterogeneity risk, which is reflected in the sensitive coefficient $\gamma_{i}$ , which is significantly positive at the 1% level. Second, the impact of the direction and intensity of common shocks themselves is reflected in the shock sensitivity coefficient $\phi_{i}$ . The negative but insignificant $\phi_{i}$ suggests that market expectation bias is the main way in which common shocks affect heterogeneity risk.

**Table 3:** Parameter estimation of the EGARCH model.

	$\alpha_{0}$	$\alpha_{1}$	$\omega_{i}$	$\gamma_{i}$	$\phi_{i}$	$\beta_{i}$	$v_{i}$	$\varphi_{i}$
Market	−0.000	0.004	−0.222^**^	0.242^***^	−0.001	0.967^***^	9.670^***^	−0.139^***^
IT	−0.001^***^	0.012^***^	−0.327^**^	0.243^***^	−0.000	0.947^***^	8.120^***^	−0.203^***^
Utility	−0.000	0.008	−0.161^**^	0.245^***^	0.029	0.975^***^	6.933^***^	−0.125^***^
Health care	−0.001	0.007	−0.221^**^	0.267^***^	0.003	0.965^***^	7.953^***^	−0.174^***^
Optional consumption	−0.001	0.002	−0.218^**^	0.259^***^	0.006	0.966^***^	7.800^***^	−0.175^***^
Industry	−0.000	0.004	−0.235^**^	0.258^***^	−0.003	0.964^***^	9.745^***^	−0.181^***^
Real Estate	−0.001	0.034	−0.146^**^	0.221^***^	0.006	0.976^***^	8.386^***^	−0.093^**^
Daily consumption	0.001	−0.020	−0.296^**^	0.278^***^	0.038	0.955^***^	11.232^***^	−0.143^***^
Material	0.000	0.023	−0.187^**^	0.229^***^	−0.002	0.970^***^	13.361^***^	−0.119^***^
Telecom	0.000	−0.037	−0.043	0.101^**^	0.046	0.992^***^	4.807^***^	0.029
Energy	0.000	−0.007	−0.121^**^	0.174^***^	−0.020	0.981^***^	6.478^***^	0.065
Finance	0.000	−0.002	−0.197^*^	0.217^***^	0.024	0.970^***^	5.817^***^	0.113^**^

### 3.3. Tail volatility spillover effects across sectors

**Figure 2:**
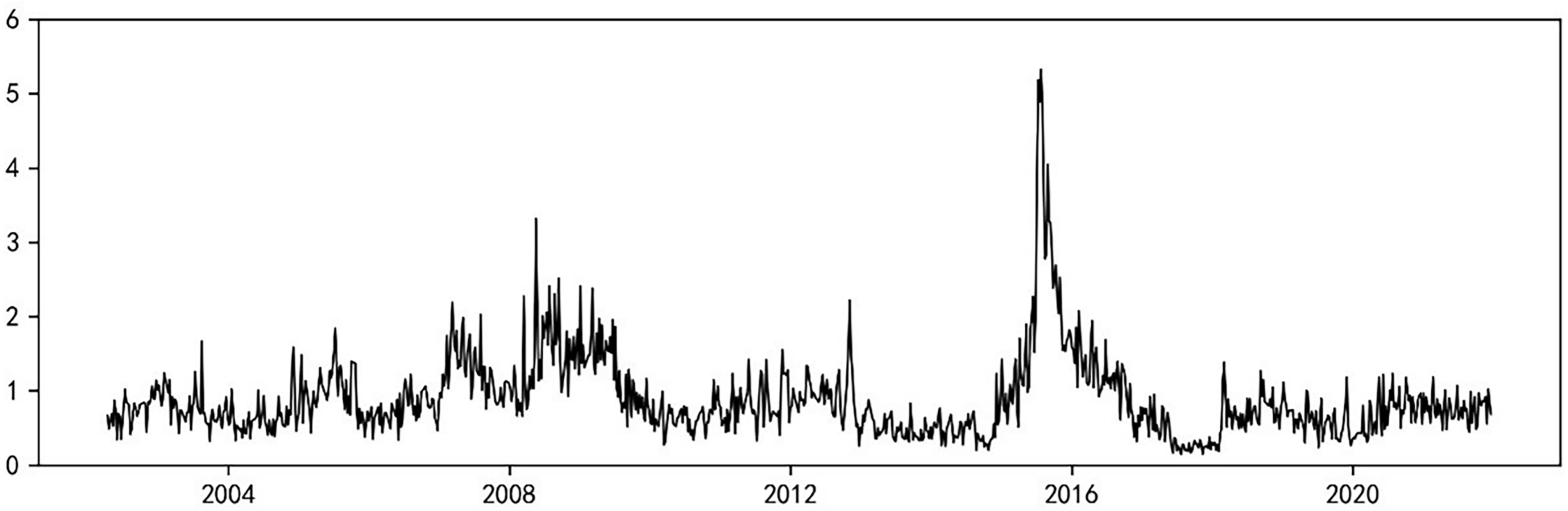
Total systemic risk spillover index across sectors.

Figure 2 reflects the total systemic risk spillover index, $SysRisk_{t}$ , calculated by combining the left-tail and right-tail risk information under a 5% tail-event (τ = 0.95) shock. As shown in Figure 2, the level of intersector systemic risk spillover in China generally shows a certain cyclicality, and the mean value of the spillover index within the sample is 0.87. Based on the LR-EGARCH-TENET model, the spillover index basically captures all major systemic risk events in the past 20 years; these events include the A-share crash caused by the spread of the subprime mortgage crisis in 2008, the risk resonance brought by the outbreak of the European debt crisis in 2012, the A-share crisis in 2015, and the market shock triggered by the trade friction between China and the U.S. in 2018. Additionally, the peak in the figure well reflects the aforementioned tail events. Overall, the two financial crises in 2008 and 2015 are more impactful and destructive, with their risk spillover index peaks reaching 3.32 and 5.32 in July 2008 and July 2015, respectively.

**Figure 3:**
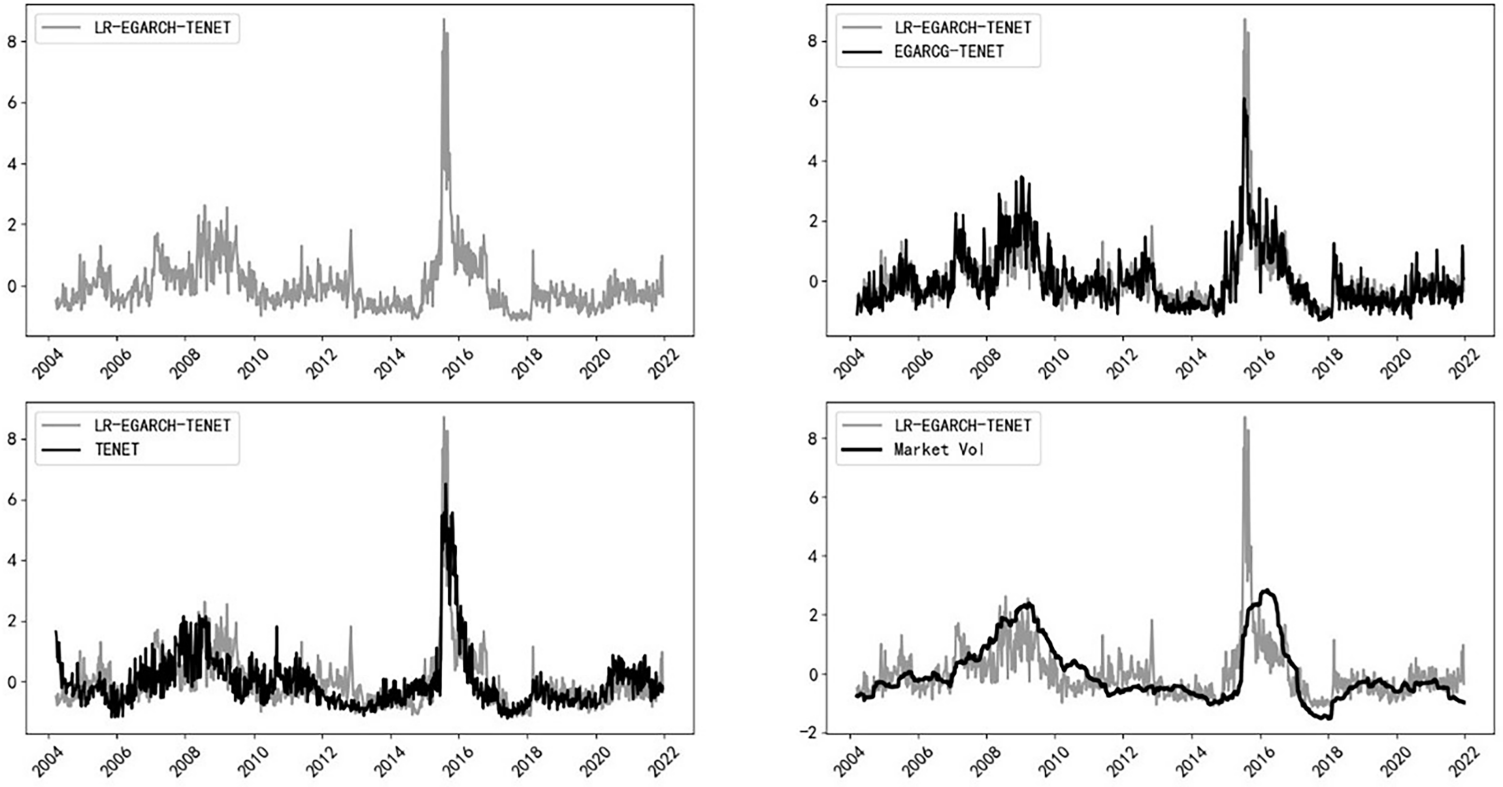
Comparison of different measurement methods.

To further compare the advantages and disadvantages of different systemic risk measures, the EGARCH-TENET model (which does not distinguish between left-tail and right-tail risks), the traditional TENET model (which is based on left-tail return losses), and stock market volatility are compared with the LR-EGARCH-TENET model constructed in this paper, and the results are reported in Figure 3. To compare various measures in the same dimension, all indicators are normalized. As shown in Figure 3, compared with the other three types of measures, the LR-EGARCH-TENET model (which distinguishes between left-tail and right-tail risks) has a higher sensitivity to extreme events, and the peak level of risk spillover is significantly larger than that obtained by the other measures when the overall trends are similar. Thus, the method is better at identifying extreme tail events while ensuring robustness.

Table 4 reports the levels of total systemic risk, left-tail risk, and right-tail risk spillover effects. According to the table, the top three tail-risk spillover levels for finance, daily consumption, and energy are systemically important sectors; this finding is consistent with the reality that the financial sector remains the largest systemic risk exporter, while information technology, real estate, and daily consumption are the three largest systemic risk takers and are systemically vulnerable sectors. The left-tail risk is similar to the total systemic risk, with finance being the systemically important sector with the largest level of risk spillover and real estate being the systemically vulnerable sector with the largest level of risk input. In the right-tail risk spillover level ranking, daily consumption is the largest risk exporter, while finance ranks only ninth in risk spillover level, suggesting that the main way the financial sector causes systemic risk is by causing immediate direct losses rather than implied asset bubbles.

**Table 4:** Level of total cross-sector systemic risk spillover.

Total			Left-tail			Right-tail		
Rank	Output	Input	Rank	Output	Input	Rank	Output	Input
Finance	107.36	77.10	Finance	117.55	76.64	Daily consumption	137.29	75.28
Daily consumption	105.58	78.47	Daily consumption	110.89	74.19	Energy	96.07	69.39
Energy	101.56	81.26	Energy	104.57	81.08	Optional consumption	83.12	69.27
Real estate	82.42	99.83	Optional consumption	78.75	73.14	Real estate	82.52	94.86
Optional consumption	78.91	77.95	Real estate	78.03	80.59	Industry	73.56	72.24
Industry	75.33	71.94	Industry	75.52	100.87	IT	72.67	92.39
Material	66.15	74.41	Material	66.12	77.60	Material	66.17	77.86
IT	64.86	104.85	Utility	65.45	79.61	Utility	52.18	69.49
Utility	63.39	78.11	Health care	59.07	78.37	Finance	49.53	66.18
Health care	56.67	71.21	IT	56.67	107.67	Health care	48.23	69.42
Telecom	56.38	43.47	Telecom	54.14	42.83	Telecom	38.35	43.30

All sectors in the table are listed in descending order of total spillover levels, and Equation 13 is summed horizontally with $J_{j}$ as the level of risk input and vertically with $I_{j}$ as the level of risk spillover. The left-tail risk represents the potential loss from sharp downward fluctuations in returns, and the right-tail risk represents the possibility of left-tail loss after the bubble burst implied by sharp upward fluctuations in returns.

**Figure 4:**
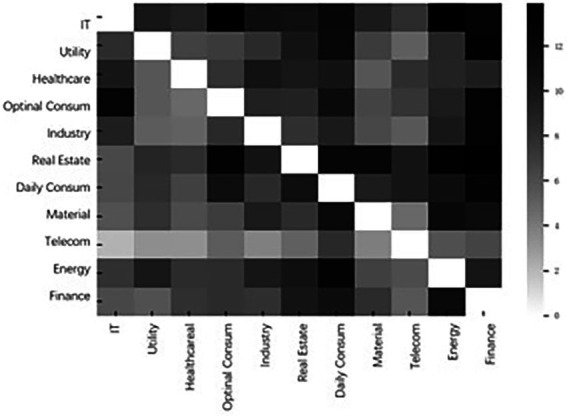
Heatmap of total systemic risk contagion.

Figure 4 is a heatmap of the cumulative interindustry systemic risk contagion network over the period of 2000–2022. Darker colors represent higher contagion intensity. The heatmap is a dediagonalized matrix, with each cell element representing the tail-risk spillover effect of the vertical coordinate sector on the horizontal coordinate sector. The horizontal sum represents the overall level of risk inputs to the diagonal industry from other industries, reflecting the systemic vulnerability of the industry. The vertical sum represents the overall level of risk spillover from the diagonal industry to other industries, reflecting the systemic importance of the industry. The information reflected in this figure is highly consistent with Table 1, where financials, daily consumption, and energy are the three largest systemically important industries in the stock market and exist primarily as sources of systemic risk in the system. In addition, information technology, real estate, and everyday consumption are the three largest systemically vulnerable sectors in the market and are susceptible to heterogeneous risk shocks from almost all other sectors.

**Figure 5:**
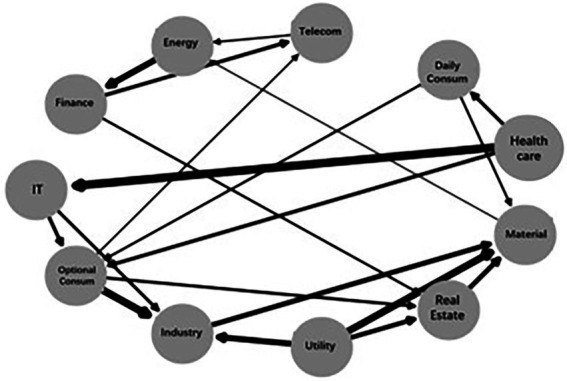
Directed acyclic graph for total systemic risk contagion.

Figure 5 investigates the causal significance and the association strength across sectors by using a directed acyclic graph. The direction of the arrow represents the significance of risk spillover between sectors, while the width of the arrow denotes the size of the Fisher z-statistic. As the figure shows, there is a significant risk spillover effect of finance, optional consumption, and utilities on real estate and a significant risk spillover effect of information technology, daily consumption, and health care on optional consumption.

Table 5 reflects the level of risk output, risk input, and the net risk spillover between any two sectors. The results show that finance, daily consumption, and energy are the top 3 industries in terms of net risk premiums, with values of 30.26, 27.11, and 20.30, respectively. Additionally, information technology, real estate, and utility are the bottom 3 industries with net premiums of −39.99, −17.41, and − 14.72, respectively.

**Table 5:** Intersector total systemic risk contagion network.

	IT	Utility	Health care	Optional consumption	Industry	Real estate	Daily consumption	Material	Telecom	Energy	Finance	From
IT	0.00	9.00	7.85	12.76	10.47	10.01	11.46	9.07	7.37	14.75	12.13	104.85
Utility	7.36	0.00	6.36	6.16	6.72	9.30	11.76	6.19	4.20	8.31	11.76	78.12
Health care	7.85	4.79	0.00	6.48	7.37	7.89	9.38	4.77	5.25	9.02	8.41	71.21
Optional consumption	10.11	5.40	4.10	0.00	7.30	8.05	11.33	5.60	5.40	8.98	11.68	77.96
Industry	8.41	4.83	4.27	7.62	0.00	7.00	9.24	5.14	4.09	9.12	12.21	71.94
Real estate	5.75	7.56	6.92	11.78	9.51	0.00	11.16	10.70	8.25	13.01	15.20	99.84
Daily consumption	5.22	6.94	5.49	8.62	6.92	9.33	0.00	7.38	7.14	10.25	11.19	78.48
Material	5.02	6.95	5.06	5.89	8.17	7.36	10.86	0.00	4.01	11.22	9.89	74.42
Telecom	1.96	3.31	3.02	4.58	3.31	4.11	8.23	3.34	0.00	5.61	6.01	43.48
Energy	7.10	9.18	6.81	7.13	8.29	10.15	11.71	6.50	5.52	0.00	8.89	81.27
Finance	6.09	5.43	6.80	7.89	7.28	9.22	10.47	7.46	5.17	11.30	0.00	77.11
To	64.86	63.40	56.67	78.92	75.34	82.42	105.59	66.15	56.38	101.57	107.37	
Net spillover	−39.99	−14.72	−14.54	0.96	3.39	−17.41	27.11	−8.27	12.91	20.30	30.26	

In addition, we also compare risk contagion effects when the market faces different levels of tail-event shocks (*τ* = 0.9, 0.95, 0.99) and calculate the difference $\Delta {\hat{D}}_{sys|{\tilde{F}}_{\tau }}={\hat{D}}_{sys,\tau }-{\hat{D}}_{sys,0.5}$ . As Table 6 clearly shows, systemic risk is extremely contagious during a crisis, and $\Delta {\hat{D}}_{sys|{\tilde{F}}_{\tau }}$ of total systemic risk is 372.57 (*τ* = 0.9), 577.09 (*τ* = 0.95), and 1282.62 (*τ* = 0.99). In addition, the intensity of risk contagion increases monotonically as the magnitude of the shock deepens (10, 5, and 1% tail shocks), regardless of total systemic risk, left-tail risk, or right-tail risk. The above results fully illustrate the sensitivity of risk contagion networks to tail shocks and their intensities.

**Table 6:** Systemic risk spillovers under different tail-event shocks.

	τ=0.9		τ=0.95		τ=0.99	
	$Sys_{\tau }$	$\Delta {\hat{D}}_{sys|{\tilde{F}}_{\tau }}$	$Sys_{\tau }$	$\Delta {\hat{D}}_{sys|{\tilde{F}}_{\tau }}$	$Sys_{\tau }$	$\Delta {\hat{D}}_{sys|{\tilde{F}}_{\tau }}$
Total systemic risk	654.13	372.57	858.66	577.09	1564.18	1282.62
Left-tail risk	672.35	407.83	866.72	602.20	1655.43	1390.91
Right-tail risk	635.98	372.81	799.26	536.12	1527.20	1264.06

To further test the connection between left-tail risk and right-tail risk, we divide the 11 sectors into finance, real estate and other real economies and then examine the Granger causality of left-tail risk and right-tail risk between any two sectors.^4^ We construct a binary VAR model for stationary left-tail risk and right-tail risk, where the AIC is applied to determine the optimal lag order, and then examine the leading-lag relationship between them. For instance, $\Delta {\hat{D}}_{e|r}^{r}$ denotes the right-tail risk of other real economies conditional on real estate, and $\Delta {\hat{D}}_{e|r}^{r}\to \Delta {\hat{D}}_{e|r}^{l}$ indicates the null hypothesis that $\Delta {\hat{D}}_{e|r}^{r}$ does not cause $\Delta {\hat{D}}_{e|r}^{l}$ . As shown in Table 7, apart from the absence of Granger causality between $\Delta {\hat{D}}_{f|e}^{r}$ and $\Delta {\hat{D}}_{f|e}^{l}$ , right-tail risk is a one-way Granger cause of left-tail risk in the remaining groups. Alternatively, right-tail risk has a stronger forward-looking predictive power for left-tail risk, implying that the rapid asset price boom in the short term is indeed a great early warning indicator of financial crises (Borio and Lowe, 2002; Schularick and Taylor, 2012; Krishnamurthy and Li, 2020; Greenwood et al., 2022).

**Table 7:** Granger causality test of left-tail risk and right-tail risk.

	Lagged order	$\chi^{2}$ value	*p* value
$\Delta {\hat{D}}_{e|r}^{r}\to \Delta {\hat{D}}_{e|r}^{l}$	10	47.1219	0.0000
$\Delta {\hat{D}}_{e|r}^{l}\to \Delta {\hat{D}}_{e|r}^{r}$	10	23.7776	0.0167
$\Delta {\hat{D}}_{r|e}^{r}\to \Delta {\hat{D}}_{r|e}^{l}$	10	89.3866	0.0000
$\Delta {\hat{D}}_{r|e}^{l}\to \Delta {\hat{D}}_{r|e}^{r}$	10	18.5159	0.0853
$\Delta {\hat{D}}_{e|f}^{r}\to \Delta {\hat{D}}_{e|f}^{l}$	7	29.7353	0.0000
$\Delta {\hat{D}}_{e|f}^{l}\to \Delta {\hat{D}}_{e|f}^{r}$	7	11.436	0.1206
$\Delta {\hat{D}}_{f|e}^{r}\to \Delta {\hat{D}}_{f|e}^{l}$	8	7.9232	0.4432
$\Delta {\hat{D}}_{f|e}^{l}\to \Delta {\hat{D}}_{f|e}^{r}$	8	3.2560	0.5980
$\Delta {\hat{D}}_{r|f}^{r}\to \Delta {\hat{D}}_{r|f}^{l}$	6	17.4202	0.0078
$\Delta {\hat{D}}_{r|f}^{l}\to \Delta {\hat{D}}_{r|f}^{r}$	6	12.4199	0.0532
$\Delta {\hat{D}}_{f|r}^{r}\to \Delta {\hat{D}}_{f|r}^{l}$	6	39.2114	0.0000
$\Delta {\hat{D}}_{f|r}^{l}\to \Delta {\hat{D}}_{f|r}^{r}$	6	20.4524	0.0235

The subscripts *e*, *r*, and f denote the real economy sector, the real estate sector, and the finance sector, respectively, while the superscripts l and r refer to left-tail risk and right-tail risk, respectively.

## 4. Conclusion

To more effectively prevent potential financial crises, we distinguish between left-tail risk and right-tail risk and then construct a tail volatility spillover network to study the accumulation, outbreak, and spillover of systemic risk in the Chinese stock market. We find that there is approximately a one-third chance that a large market decline will occur within a week following a large short-term rally. Therefore, right-tail risks that are overpriced and may result in catastrophic losses in the future merit special consideration and must be incorporated into the systemic risk measure.

When considering the effect of common shocks on the risk of heterogeneity, we find that the EGARCH model has high explanatory power. The market expectation bias of common shocks amplifies heterogeneity risk significantly. In the diffusion phase following the emergence of heterogeneity risk, we find that finance is the sector with the highest level of risk spillover, while information technology and real estate are the sectors with the highest level of risk input. We also examine the risk contagion effect when the market is exposed to varying levels of tail-event shocks, and it is evident that the risk contagion network is extremely sensitive to tail shocks and their intensity. In addition, additional research indicates that right-tail risk is a one-way Granger cause of left-tail risk. In other words, right-tail risk has a stronger forward-looking predictive power for left-tail risk, implying that the rapid rise in asset prices in the short term can be viewed as an excellent indicator of financial crises.

The regulatory insights of this paper are as follows. First, apart from the immediate regulatory objectives, Chinese supervisions should consider the existence of market expectation deviations, particularly when employing strong binding instruments. Even though the policy may positively affect the market, if the deviation from market expectations is too great, the policy is still likely to induce the emergence outbreak of right-tail risk. The second takeaway is to be wary of the nonnegligible right-tail risk, which is likely to cause severe and widespread damage in the future if the market is not restrained in the short-term surge process, especially in China, where retail investors dominate the stock market. Consequently, it is essential to include right-tail risk in measurements of systemic risk.

## Data availability statement

Publicly available datasets were analyzed in this study. This data can be found at: the CSMAR database (www.gtadata.com) and Wind database (www.wind.com.cn).

## Author contributions

JX contributed to the conception of the study and wrote the manuscript. QL contributed significantly to the analysis and manuscript preparation. MX contributed significantly to perform the data analyzes and wrote the manuscript. All authors contributed to the article and approved the submitted version.

## Funding

This research was supported by the Program for Young Innovative Research Team in China University of Political Science and Law (20CXTD10) and the major project of the National Social Science Foundation of China (21&ZD079).

## Conflict of interest

The authors declare that the research was conducted in the absence of any commercial or financial relationships that could be construed as a potential conflict of interest.

## Publisher’s note

All claims expressed in this article are solely those of the authors and do not necessarily represent those of their affiliated organizations, or those of the publisher, the editors and the reviewers. Any product that may be evaluated in this article, or claim that may be made by its manufacturer, is not guaranteed or endorsed by the publisher.

## Footnotes

^1^There are two principal reasons for studying the Chinese market. First, compared to the European and American markets, the Chinese stock market is dominated by retail investors, who are driven by herd behavior and are more likely to amplify market volatility. Second, since the 2008 global crisis, the Chinese stock market has experienced another more severe market crash in 2015, thereby providing more crisis samples for our study.

^2^We chose the data after 2000 because China passed its first Securities Law in July 1999; this law marked a new stage of the legalization of China’s capital market. To ensure that the law’s impact is fully priced by the market, we start with the year 2000.

^3^Other firm-specific crash risk measures, such as *ncrash*, *ncskew*, *duvol* and *minreturn* (Callen and Fang, 2013, 2015; Kim and Zhang, 2016; Andreou et al., 2017; Chang et al., 2017; Lobo et al., 2020; He et al., 2021), are used for the robustness check, and the key conclusions remain the same. All indicators are aggregated to the industry level.

^4^We divide the 11 sectors into these three sectors because during the 2008 crisis, financial derivatives (such as CDOs and CDSs) amplified the risk of subordinated bonds in the real estate market and spread the risk rapidly to the rest of the real economy. We would like to explore further the association between right-tail and left-tail risks across these three sectors.
